# Long-Term Outcomes After Percutaneous Coronary Intervention According to the High-Sensitivity C-Reactive Protein-to-Albumin Ratio in Patients With Chronic Obstructive Pulmonary Disease in China

**DOI:** 10.31083/RCM46633

**Published:** 2026-04-17

**Authors:** Yueyue Cao, Yicheng Tong, Zhao Wang, Yu Qi, Jun Gao, Wenyao Wang, Yi-Da Tang, Yitian Zheng

**Affiliations:** ^1^Department of Cardiology and Institute of Vascular Medicine, Peking University Third Hospital, 100191 Beijing, China; ^2^Beijing Key Laboratory of Clinical Evaluation of Cardiovascular-Kidney-Metabolic and Immuno-Inflammatory Innovative Drugs and Medical Devices, Peking University, 100191 Beijing, China; ^3^State Key Laboratory of Vascular Homeostasis and Remodeling, Peking University, 100191 Beijing, China; ^4^Research Unit of Medical Science Research Management/Basic and Clinical Research of Metabolic Cardiovascular Diseases, Chinese Academy of Medical Sciences, 100191 Beijing, China; ^5^University of Health and Rehabilitation Sciences, 266000 Qingdao, Shandong, China

**Keywords:** cardiovascular diseases, coronary artery disease, pulmonary disease, chronic obstructive, percutaneous coronary intervention, C-reactive protein, albumins

## Abstract

**Background:**

Chronic obstructive pulmonary disease (COPD) and coronary artery disease (CAD) frequently occur together, with systemic inflammation linking these two conditions. Recently, the high-sensitivity C-reactive protein to albumin ratio (hsCAR) has been identified as a composite biomarker of inflammation and nutrition. Thus, this study aimed to examine the prognostic value of hsCAR in patients with COPD–CAD undergoing percutaneous coronary intervention (PCI).

**Methods:**

In this cohort study, consecutive patients with COPD–CAD who underwent PCI between 2014 and 2019 were enrolled and categorized into tertiles by hsCAR values. The primary endpoint was major adverse cardiac events (MACEs), including cardiac death, target vessel revascularization (TVR), and nonfatal myocardial infarction (MI). Patients underwent a follow-up for up to 4 years, and the incidence of MACEs was compared between the hsCAR groups using Kaplan–Meier curves and Cox regression analyses.

**Results:**

A total of 262 patients were enrolled. Over a median follow-up of approximately 4 years, higher hsCAR levels were associated with an increased incidence of MACEs. The cumulative incidence of MACEs was highest in Group C (hsCAR ≥0.079). The incidence of MACEs was significantly higher in Group C than in Group A (19.5% vs. 5.7%; hazard ratio (HR) = 3.27, 95% confidence interval (CI): 1.08–9.86; *p* = 0.035). Receiver operating characteristic (ROC) curve analysis confirmed the associated discriminatory ability (area under the curve (AUC) = 0.651; *p* = 0.004). Restricted cubic spline (RCS) analysis showed a linear increase in the risk of MACEs as the absolute value of hsCAR exceeded 0.0446. Subgroup analyses revealed consistent associations across strata, with no significant interactions.

**Conclusion:**

Elevated baseline hsCAR is an independent predictor of long-term MACEs in patients with COPD–CAD undergoing PCI. As an inexpensive and readily available biomarker, hsCAR could be used for post-PCI risk stratification to guide targeted secondary prevention in this high-risk population.

## 1. Introduction

Chronic obstructive pulmonary disease (COPD) and coronary artery disease (CAD) are prevalent chronic disorders that contribute significantly to global mortality and impose a considerable socioeconomic burden [1, 2, 3]. Individuals with COPD exhibit a heightened susceptibility to cardiovascular disease (CVD) compared to non-COPD populations [4], with CVD accounting for nearly one-third of COPD-related fatalities [5]. The frequent co-occurrence of CAD and COPD [6] underscores a bidirectional relationship, wherein COPD is linked to an increased risk of adverse cardiovascular events [7]. Beyond common risk factors such as smoking and advanced age, heightened oxidative stress and systemic inflammation are key mechanisms connecting COPD and CAD [8]. Persistent systemic inflammation in COPD promotes endothelial dysfunction, atherosclerotic plaque progression, and thrombosis [9], processes that are directly implicated in vascular stenosis and poor cardiovascular outcomes. Consequently, biomarkers related to inflammation may offer valuable insights into the cardiovascular risk and prognosis of patients with concomitant COPD and CAD.

High-sensitivity C-reactive protein (hsCRP) is a well-established marker of systemic inflammation, whereas serum albumin reflects nutritional and metabolic health. Elevated hsCRP at the time of percutaneous coronary intervention (PCI) is associated with increased 10-year all-cause mortality and myocardial infarction (MI) risk [10]. Additionally, hsCRP serves as an independent prognostic indicator of CVD risk in COPD patients [11] and predicts long-term outcomes in those with COPD–CAD undergoing PCI [12]. Similarly, hypoalbuminemia has been identified as a predictor of all-cause mortality in acute coronary syndrome [13]. The high-sensitivity C-reactive protein to albumin ratio (hsCAR), an integration of these two parameters, has recently gained attention as a novel composite biomarker with prognostic relevance across a variety of cardiovascular and non-cardiovascular conditions. For example, a study involving 1210 COPD patients indicated that an elevated CRP-to-albumin ratio (CAR) significantly predicts 5-year mortality [14]. Moreover, hsCAR demonstrates superior predictive capability for CVD incidence compared to either hsCRP or albumin alone [15]. It has also been validated as an independent prognostic marker in PCI patients [16].

Although existing evidence supports the prognostic value of hsCAR in a diverse range of diseases, its role in predicting long-term cardiac outcomes specifically in COPD–CAD patients after PCI remains unexamined. Given the pivotal role of systemic inflammation in the initiation and progression of cardiac events, hsCAR represents a promising biomarker for risk stratification in such a high-risk group. Therefore, this study was designed to evaluate the long-term prognostic significance of hsCAR in COPD–CAD patients undergoing PCI, with the aim of informing individualized follow-up care.

## 2. Materials and Methods

### 2.1 Study Design

**Fig. 1.**
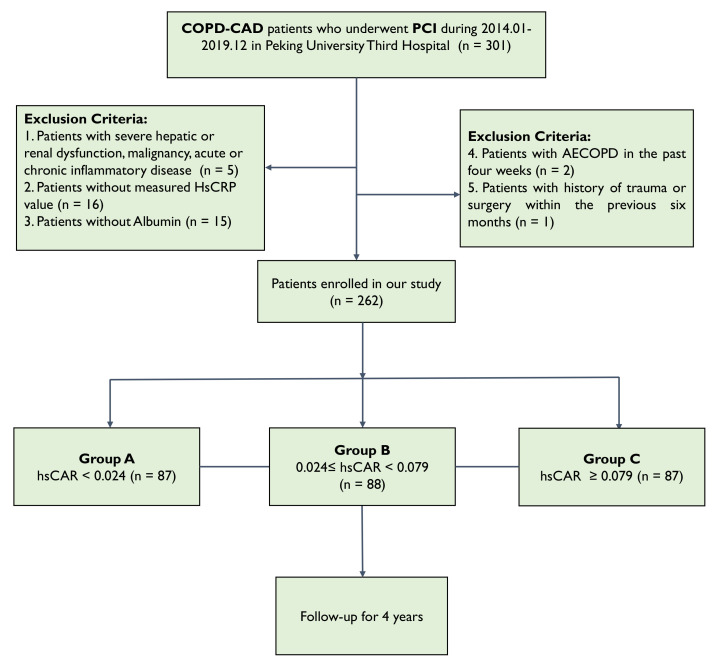
**Flowchart of study**. COPD, chronic obstructive pulmonary disease; CAD, coronary artery disease; PCI, percutaneous coronary intervention; AECOPD, acute exacerbation of chronic obstructive pulmonary disease; HsCRP, high-sensitivity C-reactive protein; hsCAR, high-sensitivity C-reactive protein to albumin ratio.

Between January 1, 2014, and December 31, 2019, we enrolled consecutive patients diagnosed with COPD–CAD who underwent PCI at Peking University Third Hospital. Laboratory tests and data collection were conducted on admission and before PCI. The enrollment process is shown in Fig. 1.

### 2.2 Participants

All participants received PCI at our institution. Inclusion criteria were: (1) diagnosis of CAD and receipt of PCI at the study hospital; (2) age ≥18 years; and (3) confirmed diagnosis of COPD.

Exclusion criteria included: (1) severe hepatic or renal impairment, malignancy, or acute/chronic inflammatory diseases; (2) missing hsCRP or serum albumin data; (3) history of acute exacerbation of COPD within the preceding 4 weeks; or (4) history of trauma or surgical procedure within the previous 6 months.

### 2.3 Endpoints

The primary endpoint was the occurrence of major adverse cardiac events (MACEs), defined as a composite of cardiac death, target vessel revascularization (TVR), or nonfatal MI within 4 years after PCI.

A secondary endpoint consisted of the composite of MACE and all-cause mortality. Cardiac death was defined per established guidelines as any death not clearly attributable to a noncardiac cause [17]. TVR was defined as recurrent angina or ischemia related to the target vessel necessitating repeat revascularization via PCI or coronary artery bypass grafting [18]. Nonfatal MI was defined as type 1 MI occurring post-PCI.

All clinical endpoints were adjudicated by an independent follow-up committee composed of at least two cardiologists who were blinded to the hsCAR grouping. In cases of disagreement among the reviewers, the final decision was rendered by Professor Yida Tang.

### 2.4 Definitions

COPD was diagnosed according to the Global Initiative for Chronic Obstructive Lung Disease (GOLD) spirometric criteria [19]. CAD was diagnosed based on coronary angiography findings. COPD severity was evaluated using GOLD spirometric staging, and coronary disease complexity was assessed by identifying multivessel disease based on coronary angiography records. Other clinical characteristics were defined and diagnosed according to the International Classification of Diseases, Ninth Revision (ICD-9) criteria.

### 2.5 Laboratory Measurements

Certified laboratory technicians, blinded to all clinical information, performed the laboratory analyses using standardized assays on automated platforms. The assessments included differential complete blood counts, comprehensive metabolic panels, and a range of specialized cardiovascular biomarkers. Measurements such as platelet count, hemoglobin concentration, serum albumin, renal function parameters, and cardiac biomarkers were obtained and are summarized in Table 1. In addition, hsCRP was quantified using the latex-enhanced immunoturbidimetric test by Beckman (Beckman Coulter Inc., Brea, CA, USA).

**Table 1.** **Baseline characteristics according to different hsCAR groups**.

Variables			Group A: hsCAR <0.024 (n = 87)	Group B: 0.024 ≤ hsCAR < 0.079 (n = 88)	Group C: hsCAR ≥0.079 (n = 87)	*p* value
Demographic characteristics						
	Age, years		69.3 ± 7.2	68.9 ± 7.8	65.2 ± 10.1	0.750
	Male, %		83.9	81.8	83.9	0.913
	CAD, %					
		CCS	21.8	36.4	36.8	0.050*
		ACS	78.2	63.6	63.2	-
Coexisting conditions, %						
	Hypertension		55.2	55.7	54.0	0.975
	Dyslipidemia		43.7	40.9	35.6	0.546
	Diabetes Mellitus		27.6	34.1	28.7	0.606
	Renal Dysfunction		4.6	11.4	4.6	0.124
	Ever Smoker		55.2	70.5	72.4	0.031*
	Current Smoker		29.9	45.5	55.2	0.003*
	Cerebrovascular Diseases		6.9	11.4	5.7	0.351
	Previous MI		9.2	2.3	4.6	0.117
	Previous CABG		0	0	0	-
	Previous PCI		0	0	1.7	-
	Peripheral Vascular Diseases		3.4	1.1	3.4	0.548
Lab test						
	HsCRP, mg/L		0.53 ± 0.24	1.83 ± 0.66	20.9 ± 27.9	<0.0001*
	Albumin, g/L		42.5 ± 3.6	40.7 ± 3.3	39.6 ± 3.6	<0.0001*
	TG, mmol/L		1.4 ± 0.6	1.6 ± 0.9	1.7 ± 1.2	0.091
	TC, mmol/L		4.0 ± 1.2	4.0 ± 1.0	4.2 ± 1.0	0.165
	HDL-C, mmol/L		1.1 ± 0.3	1.0 ± 0.3	1.0 ± 0.3	0.031*
	LDL-C, mmol/L		2.4 ± 0.9	2.5 ± 0.9	2.7 ± 0.8	0.056
	Lp(a), mmol/L		181.4 ± 246.7	186.6 ± 202.9	196.1 ± 228.3	0.911
	Urine acid, µmol/L		348.3 ± 87.3	359.8 ± 83.0	348.7 ± 92.8	0.613
	HbA1C, %		6.5 ± 1.2	6.6 ± 1.4	6.6 ± 1.4	0.962
	White blood cell, ×10^9^/L		6.0 ± 1.3	7.8 ± 2.8	8.9 ± 3.7	<0.0001*
	Neutrophil, ×10^9^/L		3.8 ± 1.2	5.4 ± 2.6	6.3 ± 3.5	<0.0001*
	Lymphocyte, ×10^9^/L		1.6 ± 0.5	1.7 ± 0.8	1.8 ± 0.7	0.126
	Creatine, µmol/L		81.0 ± 15.3	83.0 ± 19.6	79.2 ± 18.5	0.361
	BMI		25.1 ± 3.3	25.0 ± 3.0	25.0 ± 3.0	0.965
	LVEF, %		69.3 ± 7.2	68.9 ± 7.9	65.2 ± 10.2	0.029*
Angiographic and procedural details						
	Left Main involved, %		8.0	1.1	8.0	0.075
	LAD involved, %		64.4	61.4	60.9	0.878
	RCA involved, %		40.2	37.5	33.3	0.638
	LCX involved, %		28.7	26.1	29.9	0.853
	Multivessels, %		35.6	31.8	28.7	0.621
Pulmonary function test						
	FEV1/FVC, %		61.5 ± 6.5	61.9 ± 6.4	61.4 ± 10.7	0.966
	FEV1%pred, %		77.4 ± 18.1	73.8 ± 14.8	70.1 ± 19.7	0.262
	GOLD level		2.0 ± 1.0	2.5 ± 1.1	2.3 ± 1.0	0.018*
Medicine at discharge						
	β-blocker		54.0	53.4	63.2	0.342
	ACEI/ARB		11.5	22.7	23.0	0.088
	Statin		52.9	55.7	43.7	0.252
	Anti-platelet		67.8	76.1	62.1	0.130
	Bronchodilator		57.5	68.2	67.8	0.245
	Inhaled Glucocorticoid		17.2	25.0	23.0	0.435

*: *p* < 0.05. hsCAR, high-sensitivity C-reactive protein to albumin ratio; hsCRP, high-sensitivity C-reactive protein; CAD, Coronary Artery Disease; CCS, chronic coronary syndrome; ACS, acute coronary syndrome; CABG, coronary artery bypass graft; PCI, percutaneous coronary intervention; TG, triglycerides; TC, total cholesterol; HDL-C, high-density lipoprotein cholesterol; LDL-C, low-density lipoprotein cholesterol; Lp(a), lipoprotein a; HbA1C, hemoglobin A1C; LVEF, left ventricular ejection fraction; LAD, left anterior descending; RCA, right coronary artery; LCX, left circumflex; FEV1, forced expiratory volume in one second; FVC, forced vital capacity; GOLD, Global Initiative for Chronic Obstructive Lung Disease; ACEI, angiotensin converting enzyme inhibitors; ARB, angiotensin receptor blockers; BMI, body mass index.

### 2.6 PCI Procedure

All enrolled patients with COPD–CAD underwent elective PCI at our center. The specific procedural approach to PCI was determined at the discretion of the operator. Patients received standard antiplatelet therapy and heparin administration during PCI according to the patient’s bleeding risk.

### 2.7 Follow-Up

Patients were followed at 30 days, 6 months, 12 months, and annually thereafter for a total of 4 years. The final follow-up was completed in July 2021. Follow-up assessments were conducted by physicians via telephone or during outpatient clinic visits. Patients lost to follow-up were recorded as such, with the last contact date noted. For time-to-event analyses, individuals who did not complete the follow-up visit were treated as censored data.

### 2.8 Data Collection

Clinical data, including demographic information and laboratory results, were extracted from the hospital electronic medical records by an investigator blinded to the study objectives, so as to minimize information bias. hsCAR was computed as the ratio of hsCRP (mg/L) to serum albumin (g/L). Based on baseline hsCAR values, patients were divided into three groups: Group A (hsCAR <0.024), Group B (hsCAR 0.024–0.079), and Group C (hsCAR ≥0.079).

### 2.9 Statistical Analysis

Sample size calculation was performed using the G*Power 3.1 software (Düsseldorf, Germany), assuming a two-sided α of 0.05, a statistical power of 0.80, and an anticipated hazard ratio (HR) of 3.0 between patients with high versus low inflammatory response, based on previous findings [12]. Continuous variables are expressed as mean ± standard deviation and compared using the Student’s *t*-test or Mann-Whitney U test, as appropriate. Categorical variables are presented as counts and percentages and compared using the chi-square test. Multiple imputation was used to handle missing data. Specifically, we used the fully conditional specification (FCS) approach with the multivariate imputation by chained equations (MICE) algorithm, incorporating hsCAR and relevant covariates (e.g., age, sex, body mass index (BMI), hypertension, diabetes, smoking status, lipid profile).

Collinearity analysis was conducted to evaluate correlations between hsCAR and other variables. Variables with a variance inflation factor (VIF) >5–10 were considered to indicate moderate to significant collinearity, reflecting the relationship between variables and hsCAR.

Time-to-event outcomes were analyzed using Kaplan–Meier curves with log-rank tests. Multivariable Cox proportional hazards regression models were employed to adjust for confounders. HRs and 95% confidence intervals (CIs) were estimated using the Mantel-Cox method.

Receiver operating characteristic (ROC) curve analysis, Hosmer-Lemeshow goodness-of-fit test, and decision curve analysis (DCA) were used to assess the discriminative ability of hsCAR for predicting MACE.

Restricted cubic spline (RCS) analysis was conducted to explore the relationship between hsCAR and the HR. Subgroup analyses were performed to assess the homogeneity of effects among different COPD–CAD subgroups. *p* values for interaction were calculated to determine whether significant interactions were present between hsCAR and subgroup variables.

All statistical analyses and plotting of figures were performed using SPSS version 26.0 (IBM Corp., Armonk, NY, USA), GraphPad Prism version 8.0 (GraphPad Software, San Diego, CA, USA), and RStudio version 4.0 (Posit Team, Boston, MA, USA). *p* values < 0.05 were considered statistically significant.

## 3. Results

### 3.1 Baseline Characteristics

A total of 262 patients with COPD–CAD who underwent PCI at our center from January 2014 to December 2019 were included in the study, with 87, 88, and 87 patients in Groups A, B, and C, respectively. The mean follow-up duration was 4 years. Among the 262 enrolled patients, 15 patients (5.7%) were lost to follow-up.

The baseline characteristics are shown in Table 1. Patients with a higher level of hsCAR had a higher prevalence of chronic coronary syndrome (21.8, 36.4, and 36.8% in Groups A, B, and C, respectively; *p* = 0.05), a higher prevalence of current smoking (29.9, 45.5, and 55.2% in Groups A, B, and C, respectively; *p* = 0.003), and a higher prevalence ever smoking (55.2, 70.5, and 72.4 in Groups A, B, and C, respectively; *p* = 0.031). Patients with a higher level of hsCAR also had lower high-density lipoprotein cholesterol levels (1.1, 1.0, and 1.0 mmol/L in Groups A, B, and C, respectively; *p* = 0.031), higher white blood cell counts (6.0, 7.8, and 8.9 × 10^9^/L in Groups A, B, and C, respectively; *p* < 0.001), higher neutrophil counts (3.8, 5.4, and 6.3 × 10^9^/L in Groups A, B, and C, respectively; *p* < 0.001), and lower left ventricular ejection fraction (69.3, 68.9, and 65.2% in Groups A, B, and C, respectively; *p* = 0.029). The GOLD level showed significant differences among the three groups (2.0, 2.5, and 2.3 in Groups A, B, and C, respectively; *p* = 0.018). The other baseline characteristics did not differ significantly between the three groups.

### 3.2 Collinearity Analysis

The results of the collinearity analysis are presented in Table 2. The VIF values revealed that hsCAR was highly correlated with male sex (VIF: 10.23); total cholesterol (VIF: 217.51), high-density lipoprotein cholesterol levels (VIF: 11.57), low-density lipoprotein cholesterol levels (VIF: 171.75), and white blood cell (VIF: 573.69), neutrophil (VIF: 508.96), and lymphocyte (VIF: 65.95) counts. The triglyceride level was also correlated with hsCAR (VIF = 7.25). Other variables, such as gender, pulmonary function, age, and drug use, etc. also presented close relationship with hsCAR, indicating the potential value of hsCAR in reflecting worse inflammation and metabolism status.

**Table 2.** **Co-linearity analysis of baseline variables and hsCAR**.

Variables		Unstandardized coefficients		Coefficients	*t*	Sig.	Collinearity statistics	
		B	Std. error	Beta			Tolerance	VIF
Constant		0.41	0.26		1.56	0.141		
	HsCRP	0.03	0.00	0.99	27.78	0.000	0.13	7.84
	Albumin	–0.01	0.01	–0.05	–1.25	0.232	0.11	8.81
Demographic characteristics								
	Age	0.00	0.02	0.00	0.01	0.996	0.17	5.75
	Male	–0.01	0.06	–0.01	–0.24	0.811	0.10	10.23
	CCS	0.01	0.03	0.01	0.29	0.776	0.16	6.17
Coexisting conditions								
	Hypertension	0.01	0.03	0.01	0.22	0.828	0.24	4.24
	Dyslipidemia	0.03	0.03	0.02	0.99	0.341	0.26	3.85
	Diabetes Mellitus	–0.06	0.03	–0.06	–2.00	0.065	0.18	5.53
	Renal Dysfunction	0.07	0.10	0.03	0.72	0.486	0.11	8.73
	Ever Smoker	0.01	0.03	0.01	0.43	0.673	0.16	6.30
	Current Smoker	–0.01	0.03	–0.01	–0.48	0.637	0.17	5.77
	Cerebrovascular Diseases	–0.01	0.04	–0.01	–0.19	0.849	0.20	5.05
	Previous MI	–0.02	0.03	–0.01	–0.48	0.636	0.35	2.89
	Peripheral Vascular Diseases	0.07	0.06	0.03	1.26	0.227	0.34	2.93
Lab test								
	TG	0.03	0.02	0.04	1.21	0.245	0.14	7.25
	TC	–0.11	0.09	–0.22	–1.17	0.261	0.00	217.51
	HDL-C	0.00	0.08	0.00	–0.04	0.968	0.09	11.57
	LDL-C	0.15	0.11	0.23	1.38	0.190	0.01	171.75
	Lp(a)	0.00	0.00	0.00	0.11	0.912	0.17	5.98
	Urine acid	0.00	0.00	–0.01	–0.34	0.738	0.17	5.81
	HbA1C	0.00	0.01	0.01	0.46	0.650	0.21	4.69
	White blood cell	–0.01	0.06	–0.07	–0.22	0.832	0.00	573.69
	Neutrophil	0.00	0.06	–0.01	–0.03	0.980	0.00	508.96
	Lymphocyte	–0.01	0.08	–0.02	–0.16	0.877	0.02	65.95
	Creatine	0.00	0.00	0.00	0.07	0.942	0.16	6.17
	LVEF	0.00	0.00	–0.05	–1.40	0.183	0.15	6.81
Angiographic and procedural details								
	Left Main involved	–0.04	0.05	–0.02	–0.74	0.469	0.24	4.17
	LAD involved	0.01	0.03	0.01	0.54	0.600	0.24	4.25
	RCA involved	–0.06	0.03	–0.06	–1.83	0.089	0.16	6.39
	LCX involved	–0.03	0.03	–0.04	–1.36	0.194	0.24	4.10
	FEV1/FVC	0.00	0.00	0.02	0.43	0.671	0.06	15.55
	FEV1%pred	0.00	0.00	0.05	1.20	0.249	0.08	12.03
	GOLD level	–0.01	0.05	–0.02	–0.24	0.813	0.04	26.75
Medicine at discharge								
	β-blocker	0.03	0.03	0.04	1.15	0.268	0.18	5.71
	ACEI/ARB	–0.03	0.04	–0.02	–0.73	0.480	0.16	6.43
	Statin	0.00	0.02	0.00	–0.12	0.908	0.35	2.89
	Anti-platelet	–0.05	0.04	–0.04	–1.03	0.321	0.10	10.23
	Bronchodilator	0.04	0.04	0.04	0.94	0.364	0.08	12.54
	Inhaled Glucocorticoid	–0.01	0.06	–0.01	–0.25	0.810	0.09	10.88

*: *p* < 0.05. CAD, Coronary Artery Disease; CCS, chronic coronary syndrome; CABG, coronary artery bypass graft; PCI, percutaneous coronary intervention; TG, triglycerides; TC, total cholesterol; HDL-C, high-density lipoprotein cholesterol; LDL-C, low-density lipoprotein cholesterol; Lp(a), lipoprotein a; HbA1C, hemoglobin A1C; LVEF, left ventricular ejection fraction; LAD, left anterior descending; RCA, right coronary artery; LCX, left circumflex; TVD, three vessel disease; FEV1, forced expiratory volume in one second; FVC, forced vital capacity; GOLD, Global Initiative for Chronic Obstructive Lung Disease; ACEI, angiotensin converting enzyme inhibitors; ARB, angiotensin receptor blockers; MI, myocardial infarction.

### 3.3 Primary and Secondary Endpoints

**Fig. 2.**
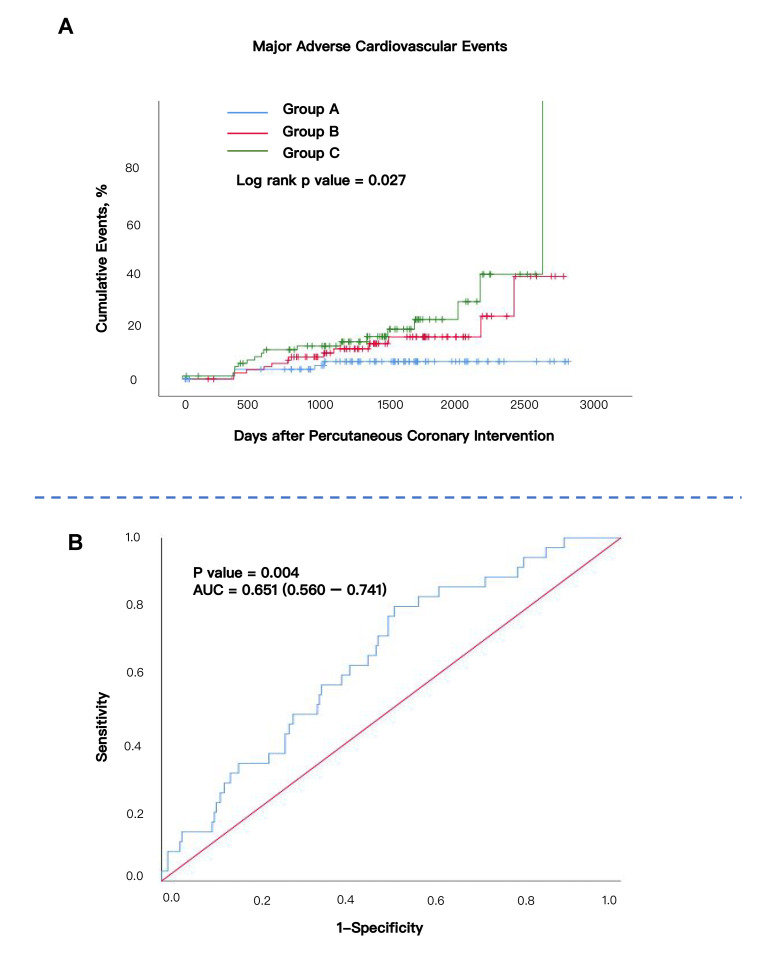
**Kaplan–Meier Curve according to two tertiles and AUC curve**. (A) Kaplan–Meier Curve according to three tertiles. Log rank *p* value = 0.027. (B) ROC curve of hsCAR in COPD–CAD participants. AUC = 0.651 (0.560–0.741). *p* value = 0.004. AUC, area under the curve; ROC, receiver operating characteristic.

The Kaplan–Meier curves of the incidence of MACE in the three groups are shown in Fig. 2A. Group C shows the highest MACE event rate compared to Groups A and B (log rank *p* value = 0.027). The ROC analysis showed that hsCAR had moderate predictive value for the occurrence of MACE after PCI (area under the curve [AUC] = 0.651, 95% CI: 0.560–0.741, *p* = 0.004) (Fig. 2B). The Hosmer-Lemeshow test yielded a *p*-value of 0.139, indicating that the model demonstrates a good fit to the data. The DCA showed that the clinical utility curve of the model lies above the “treat all” and “treat none” reference lines across a reasonable threshold probability range, suggesting meaningful clinical net benefit (**Supplementary Fig. 1**).

A comparison of the incidence of primary and secondary endpoints in the three groups (Table 3) showed that the incidence of MACE was significantly higher in Group C than in Group A (19.5% vs. 5.7%; HR = 3.27, 95% CI: 1.08–9.86; *p* = 0.035). TVR appeared to be the main contributor to the differences between groups in the incidence of MACE (*p* = 0.039). Among patients who developed TVR, repeat PCI was the predominant revascularization strategy, whereas only two cases underwent coronary artery bypass grafting (approximately 6% of all TVR events). This distribution is consistent with contemporary clinical data showing that coronary artery bypass grafting is infrequently selected for in-stent restenosis after PCI when PCI remains technically feasible [20]. No cases of cardiac death or MI were observed in Group A, whereas two cases occurred in both Groups B and C. The other endpoints did not differ significantly between groups. We also conducted Cox regression analysis using different models to confirm the robustness of hsCAR in predicting MACEs. As shown in **Supplementary Table 1**, the MACE remains higher in Group C than in Group A, in which model (All *p* < 0.05, **Supplementary Table 1**).

**Table 3.** **Long-term outcomes according to HsCAR levels before and after multivariate Cox regression adjustment**.

Endpoints			No. of Events (%)	Hazard Ratio (95% confidence interval)	*p* value	Adjusted Hazard Ratio (95% confidence interval)	Adjusted *p* value
Primary Endpoint							
	MACE						
		Group A: hsCAR <0.024	5 (5.7)	ref	ref	ref	ref
		Group B: 0.024 ≤ hsCAR < 0.079	13 (14.8)	2.56 (0.91–7.18)	0.075	2.70 (0.92–7.95)	0.071
		Group C: hsCAR ≥0.079	17 (19.5)	3.66 (1.35–9.92)	0.011*	3.27 (1.08–9.86)	0.035*
Secondary Endpoint							
	TVR						
		Group A: hsCAR <0.024	5 (5.7)	ref	ref	ref	ref
		Group B: 0.024 ≤ hsCAR < 0.079	11 (12.5)	2.17 (0.75–6.24)	0.152	2.25 (0.74–6.79)	0.151
		Group C: hsCAR ≥0.079	17 (19.5)	3.65 (1.34–9.91)	0.011*	3.21 (1.06–9.74)	0.039*
	Cardiac Death/MI						
		Group A: hsCAR <0.024	0	ref	ref	ref	ref
		Group B: 0.024 ≤ hsCAR < 0.079	2 (2.3)	–	–	–	–
		Group C: hsCAR ≥0.079	2 (2.3)	–	–	–	–
	All-cause death						
		Group A: hsCAR <0.024	4 (4.6)	ref	ref	ref	ref
		Group B: 0.024 ≤ hsCAR < 0.079	2 (2.3)	0.55 (0.10–3.02)	0.49	0.57 (0.10–3.56)	0.545
		Group C: hsCAR ≥0.079	4 (4.6)	1.30 (0.32–5.28)	0.712	1.44 (0.26–8.06)	0.681

*: *p* < 0.05. Confounding factors included in the multivariate Cox regression model: TG, TC, HDL-C, LDL-C, White blood cell, and Neutrophil. MACEs, major adverse cardiac events; TVR, target vessel revascularization.

### 3.4 RCS and Subgroup Analysis

**Fig. 3.**
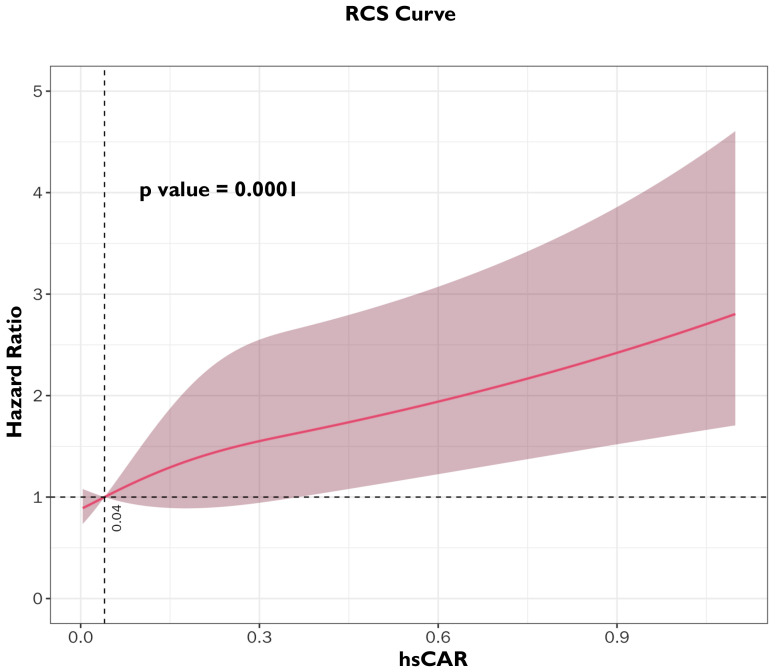
**Restricted cubic spline (RCS) between hsCAR and Hazard ratio in COPD–CAD participants**.

To further investigate the association between hsCAR and the risk of MACE after PCI in patients with COPD–CAD, an RCS analysis was performed; the results are shown in Fig. 3. The RCS curve showed that the HR for MACE increased progressively with higher levels of hsCAR (*p* < 0.0001). When hsCAR exceeded 0.0446, the HR for MACE increased over 1.0.

Subgroup analyses showed that the risk of MACE was higher in Group C than in Group A (HR >1.00) in all subgroups analyzed, including subgroups based on age, sex, current smoking status, ever smoking status, hypertension, and dyslipidemia (Table 4). The differences between groups, however, were statistically significant (*p* < 0.05) only in male patients, non-current smokers, ever smokers, and patients with hypertension. No significant interactions were detected between hsCAR and any of the subgroup variables (all *p* for interaction >0.05).

**Table 4.** **Summary table of subgroup analysis**.

Subgroup		No. of Patients	MACEs (%)	Non-MACEs (%)	HR for Group C refers to Group A (95% CI)	*p* value	*p* value for Interaction
Overall		262	35 (13.4)	227 (86.6)	3.66 (1.35–9.92)	0.011*	
Age, year							
	<65	141	19 (13.5)	122 (86.5)	2.26 (0.71–7.23)	0.168	0.257
	≥65	121	16 (13.2)	105 (86.8)	8.00 (0.98–65.21)	0.052	ref
Gender							
	Male	218	28 (12.8)	190 (87.2)	5.04 (1.44–17.59)	0.011*	0.338
	Female	44	7 (15.9)	37 (84.1)	1.52 (0.26–9.13)	0.644	ref
Current Smoker							
	Yes	114	16 (14.0)	98 (86.0)	2.05 (0.43–9.78)	0.369	0.327
	No	148	19 (12.8)	129 (87.2)	5.11 (1.38–18.94)	0.015*	ref
Ever Smoker							
	Yes	173	26 (15.0)	147 (85.0)	5.49 (1.21–24.94)	0.027*	0.542
	No	89	9 (10.1)	80 (89.9)	2.66 (0.64–11.17)	0.18	ref
Hypertension							
	Yes	144	21 (14.6)	123 (85.4)	6.16 (1.38–27.56)	0.017*	0.232
	No	118	14 (11.9)	104 (88.1)	1.60 (0.38–6.69)	0.521	ref
Dyslipidemia							
	Yes	105	12 (11.4)	93 (88.6)	6.48 (0.78–54.07)	0.081	0.433
	No	157	23 (14.6)	144 (91.7)	2.72 (0.87–8.45)	0.085	ref

*: *p* < 0.05. MACE, major adverse cardiovascular events; HR, hazard ratio.

## 4. Discussion

In this cohort of 262 COPD–CAD patients treated with PCI, we found that hsCAR effectively predicts long-term adverse cardiovascular outcomes. During a 4-year follow-up, higher hsCAR at PCI was associated with significantly increased cumulative MACE incidence. After multivariate adjustment, the high-hsCAR group (Group C) maintained a significantly elevated risk compared to the low-hsCAR group (Group A). RCS analysis confirmed a monotonic relationship between increasing hsCAR and MACE risk, with a consistent risk rise beyond a hsCAR threshold of 0.0446. These results underscore the prognostic utility of hsCAR in this population, supporting its potential as a practical tool for post-PCI risk assessment.

Our findings align with previous studies in broader CAD populations. For instance, a multicenter prospective cohort study demonstrated that both elevated hsCRP and hypoalbuminemia independently predict long-term mortality, with the highest risk observed in patients exhibiting both abnormalities [21]. Other studies have confirmed the prognostic value of CAR in PCI settings. A study of 1630 CAD patients undergoing PCI found significant associations between CAR levels and both all-cause and cardiac mortality, identifying CAR as an independent predictor of these outcomes [22]. Additionally, hsCAR independently predicts MACE and MI in CAD patients receiving drug-eluting stents [16] and holds prognostic value in patients with ST-elevation MI undergoing primary PCI [23].

Studies focusing on PCI patients with different comorbidities have reported similar findings. In a prospective observational cohort study of 2755 patients with type 2 diabetes mellitus treated with PCI and dual antiplatelet therapy, higher CAR levels were associated with worse 5-year outcomes [24]. In patients with chronic total occlusion undergoing PCI, incorporating hsCAR into conventional risk prediction models significantly improved their prognostic accuracy [25]. Collectively, these consistent findings confirm that hsCAR is a robust predictor of post-PCI outcomes in patients with different types of CAD and different comorbidities. Our study extends these findings to patients with COPD–CAD, a subgroup that has not received attention in previous research, further demonstrating the broad prognostic utility of hsCAR in diverse types of patients with CAD.

In our RCS analysis, the inflection point is at hsCAR >0.0446, above which the risk of MACE increased almost linearly. Notably, this threshold is highly consistent with previously reported hsCAR cut-offs in different cardiovascular cohorts. Yang *et al*. [16] examined hsCAR in patients undergoing PCI and reported optimal cut-off values ranging from 0.027 to 0.134 for predicting adverse cardiovascular outcomes. Similarly, Yang *et al*. [15] identified hsCAR cut-offs of 0.0267 and 0.0622 for stratifying CVD risk in a large Chinese community population. Although the exact numerical values differ, likely due to variations in study populations, baseline risk profiles, and sample sizes, these studies consistently suggest that hsCAR values in the approximate range of 0.03–0.06 are associated with increased cardiovascular risk. Our spline-derived threshold (hsCAR ≈ 0.045) falls within this range, thereby supporting the external plausibility and clinical relevance of hsCAR as a prognostic biomarker in COPD–CAD patients after PCI.

In COPD, chronic oxidative stress driven by excessive reactive oxygen species production contributes to endothelial dysfunction, lipid oxidation, and plaque instability, thereby accelerating atherosclerosis progression. Reactive oxygen species-mediated activation of nuclear factor kappa-light-chain-enhancer of activated B cells (NF-κB) and cytokine cascades (e.g., Interleukin (IL)-6, tumor necrosis factor-alpha (TNF-α)) sustains a low-grade systemic inflammation that may be reflected indirectly by elevated hsCRP and reduced albumin levels [5]. Although hsCAR integrates inflammatory and nutritional information through hsCRP and albumin, we did not include additional biomarkers such as interleukin-6, tumor necrosis factor-α, or prealbumin, which could have provided more direct evidence linking systemic inflammation and malnutrition to adverse outcomes. Future studies could integrate multi-marker panels, including oxidative stress biomarkers (e.g., malondialdehyde, superoxide dismutase), pro-inflammatory cytokines, and nutritional indices, to elucidate the mechanistic pathways linking hsCAR, COPD-related inflammation, and adverse cardiovascular outcomes.

The prognostic value of hsCAR may be attributed to its ability to simultaneously capture both inflammatory and nutritional status. While hsCRP alone is easily affected by infection and acute stress, albumin levels change more slowly and lack sensitivity. By integrating these two parameters, hsCAR amplifies their opposite biological responses and reduces the confounding effects associated with fluctuations in a single biomarker. Consequently, hsCAR provides a more stable and reliable indicator for risk prediction. Consistent with previous findings, several studies have reported that hsCAR exhibits greater prognostic accuracy for cardiovascular events compared with hsCRP or albumin alone [15, 24, 26].

Notably, although the HR of Group C versus Group A was >1.00 in all subgroups analyzed, including age, sex, smoking status, hypertension, and dyslipidemia, the difference was statistically significant only in males, non-smokers, former smokers, and patients with hypertension. This may be attributed to the influence of smoking status, sex, and related factors on systemic inflammation and nutritional reserves, which in turn affected these subgroup-specific outcomes [27, 28, 29]. It should be noted that the small sample size within each subgroup may have limited the accuracy and stability of the subgroup analyses. Nevertheless, these intergroup differences highlight the importance of considering patient characteristics, lifestyle factors, and comorbidities in future research and clinical application of hsCAR, as such variables may modify the inflammatory burden and potentially influence its predictive value in different subpopulations.

Moreover, several potential confounders were not fully adjusted for, which may have influenced our findings. For instance, although diabetes and HbA1c were included as variables in the analysis, the collinearity test did not reveal high interaction with hsCAR. This may be attributed to the limited sample size. Previous studies have demonstrated that HbA1c levels are independently associated with cardiovascular events and all-cause mortality [30, 31]. Second, long-term medication for cardioprotective or anti-inflammatory agents such as statins, β-blockers, or ACEI/ARBs could markedly change when long-term follow-up is conducted, but affect the inflammation status a lot [32]. Finally, although current/former smoking status was recorded, quantitative tobacco exposure (e.g., pack-years) was not assessed, potentially resulting in residual confounding. Future studies are encouraged to incorporate HbA1c levels, standardized medication adherence metrics, and quantitative smoking indicators to enhance the robustness of the findings.

## 5. Limitations

There are some other limitations to consider. First, as a single-center cohort in China, the generalizability of findings to other populations may be limited. Future multicenter studies with larger and more diverse populations are warranted to externally validate our findings. Second, as an observational study, despite adjustment for known confounders, the possibility of residual confounding cannot be excluded. Moreover, previous studies have shown that the inflammatory state is associated with the severity of COPD and the complexity of CVD [33, 34]. Although our study reported the GOLD level and the presence of multivessel disease in the baseline characteristics, we did not perform stratified analyses based on COPD severity or CAD complexity. Future research should incorporate refined COPD severity classification and more comprehensive coronary complexity indices (e.g., SYNTAX score) to enhance generalizability. The study did not high AUC value, and the AUC curve below 0.7 should be carefully interpreted. Considering the primary focus of our study is to provide a proof-of-concept, demonstrating the feasibility of constructing such a prediction model using real-world data, the model utilizes routinely available clinical laboratory indicators, making it suitable for rapid screening. It holds profound value in terms of feasibility and predictive capability. But more studies remain to be conducted. Finally, hsCRP and albumin were measured only once at baseline, and we did not perform a longitudinal assessment of changes in hsCAR over time during the follow-up period. Both parameters may fluctuate over time due to post-PCI inflammation, secondary infections, comorbidities, or nutritional alterations, potentially affecting the temporal stability of hsCAR and its prognostic interpretation.

Despite these limitations, our study offers clinically relevant evidence that hsCAR is an independent predictor of long-term cardiovascular events in COPD–CAD patients after PCI. The routine availability and low cost of hsCRP and albumin measurements make hsCAR a practical tool for widespread clinical use. Patients with elevated hsCAR may benefit from intensified management. Future large-scale multicenter studies are needed to validate these findings, refine the subgroup analyses, and explore whether interventions targeting hsCAR modulation—such as anti-inflammatory agents or nutritional optimization—can improve outcomes in this high-risk population. Future interventional trials investigating hsCAR-guided strategies may help clarify its causal and therapeutic implications.

## 6. Conclusions

This study demonstrated that hsCAR is a reliable predictor of long-term adverse cardiovascular outcomes in patients with COPD–CAD undergoing PCI. Elevated hsCAR levels were independently associated with an increased risk of long-term adverse cardiovascular outcomes, particularly MACE and TVR. There was a positive linear relationship between hsCAR and MACE risk, with a marked risk increase in patients with a hsCAR value above 0.0446. HsCAR may provide a practical, cost-effective tool for post-PCI risk stratification in patients with COPD–CAD. Future multicenter studies with larger cohorts are warranted to validate our findings and to explore whether targeted anti-inflammatory or nutritional interventions can improve cardiovascular prognosis by modulating hsCAR.

## Availability of Data and Materials

All supporting data and materials are available from the corresponding author upon reasonable request.

## Supplementary Material

Supplementary material associated with this article can be found, in the online version, at https://doi.org/10.31083/RCM46633.
