# Supplementary material for: Long-Term Outcomes After Percutaneous Coronary Intervention According to the High-Sensitivity C-Reactive Protein-to-Albumin Ratio in Patients With Chronic Obstructive Pulmonary Disease in China
Source: Rev Cardiovasc Med. 2026 Apr 17;27(4):46633. doi: 10.31083/RCM46633 (PMC13156000; doi:10.31083/RCM46633)
Supplement: Supplementary file 1 [file 2153-8174-27-4-46633-s1.zip › Supplementary Material.pdf]

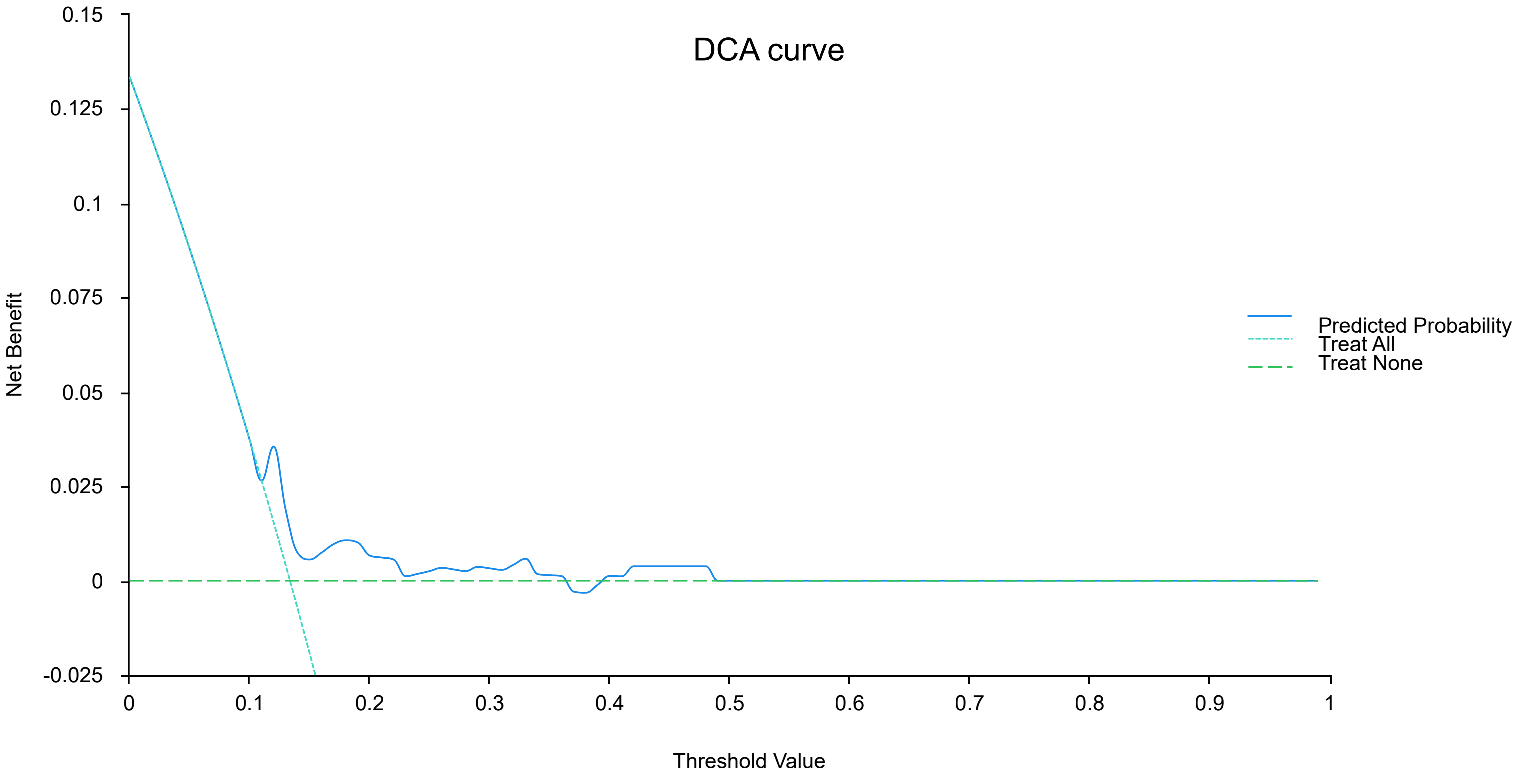

Figure S1. DCA curve of hsCAR

Table S1. Long-term Outcomes before and after different Multivariate COX Regression Models Adjustment

Endpoints	No. of Events (%)	Hazard Ratio (95% confidence interval)	P value
Before Adjustments			
**MACE**			
Group A, HsCAR < 0.024	5 (5.7)	ref	ref
Group B, 0.024<= HsCAR < 0.079	13 (14.8)	2.56 (0.91 - 7.18)	0.075
Group C, HsCAR >= 0.079	17 (19.5)	3.66 (1.35 - 9.92)	0.011*
After Adjustments			
**Model 1**			
Group A, HsCAR < 0.024	5 (5.7)	ref	ref
Group B, 0.024<= HsCAR < 0.079	13 (14.8)	2.70 (0.92 - 7.95)	0.071
Group C, HsCAR >= 0.079	17 (19.5)	3.27 (1.08 - 9.86)	0.035*
**Model 2**			
Group A, HsCAR < 0.024	5 (5.7)	ref	ref
Group B, 0.024<= HsCAR < 0.079	13 (14.8)	2.44 (0.87 - 6.89)	0.091
Group C, HsCAR >= 0.079	17 (19.5)	3.68 (1.36 - 10.0)	0.011*
**Model 3**			
Group A, HsCAR < 0.024	5 (5.7)	ref	ref
Group B, 0.024<= HsCAR < 0.079	13 (14.8)	2.24 (0.79 - 6.38)	0.131
Group C, HsCAR >= 0.079	17 (19.5)	3.36 (1.22 - 9.22)	0.019*

*: $p < 0.05$

Confounding factors included in mutivariate COX regression **Model 1**： TG, TC, HDL-C, LDL-C, White blood cell, Neutrophil; **Model 2**: Gender, Diabetes Mellitus, Hypertension, Renal dysfunction; **Model 3**: Gender, Diabetes Mellitus, Hypertension, Renal dysfunction, Smoker, Acute coronary syndrome.
